# The Impact of Due Process and Disruptions on Emergency Medicine Education in the United States

**DOI:** 10.5811/westjem.2019.10.42800

**Published:** 2020-01-27

**Authors:** Al’ai Alvarez, Anne Messman, Melissa Platt, Megan Healy, Elaine B. Josephson, Shawn London, Douglas Char

**Affiliations:** * Stanford University School of Medicine, Department of Emergency Medicine, Palo Alto, California; † Wayne State University School of Medicine, Department of Emergency Medicine, Detroit, Michigan; ‡ University of Louisville School of Medicine, Department of Emergency Medicine, Louisville, Kentucky; § Temple University Lewis Katz School of Medicine, Department of Emergency Medicine, Philadelphia, Pennsylvania; ¶ Weill Cornell Medical College of Cornell University, Lincoln Medical and Mental Health Center, Department of Emergency Medicine, Bronx, New York; || University of Connecticut School of Medicine, Department of Emergency Medicine, Farmington, Connecticut; # Washington University School of Medicine, Department of Emergency Medicine, St. Louis Missouri

## Abstract

**Introduction:**

Academic Emergency Medicine (EM) departments are not immune to natural disasters, economic or political forces that disrupt a training program’s operations and educational mission. Due process concerns are closely intertwined with the challenges that program disruption brings. Due process is a protection whereby an individual will not lose rights without access to a fair procedural process. Effects of natural disasters similarly create disruptions in the physical structure of training programs that at times have led to the displacement of faculty and trainees. Variation exists in the implementation of transitions amongst training sites across the country, and its impact on residency programs, faculty, residents and medical students.

**Methods:**

We reviewed the available literature regarding due process in emergency medicine. We also reviewed recent examples of training programs that underwent disruptions. We used this data to create a set of best practices regarding the handling of disruptions and due process in academic EM.

**Results:**

Despite recommendations from organized medicine, there is currently no standard to protect due process rights for faculty in emergency medicine training programs. Especially at times of disruption, the due process rights of the faculty become relevant, as the multiple parties involved in a transition work together to protect the best interests of the faculty, program, residents and students. Amongst training sites across the country, there exist variations in the scope and impact of due process on residency programs, faculty, residents and medical students.

**Conclusion:**

We report on the current climate of due process for training programs, individual faculty, residents and medical students that may be affected by disruptions in management. We outline recommendations that hospitals, training programs, institutions and academic societies can implement to enhance due process and ensure the educational mission of a residency program is given due consideration during times of transition.

## INTRODUCTION

Due process rights of physicians come from many sources. The legal requirement of due process in the United States (U.S.) ensures that an individual not lose rights without access to fair procedural process. In clinical practice, due process means clinicians do not lose their medical staff privileges without a fair hearing. For the specialty of Emergency Medicine (EM), residency program faculty are assigned their roles and duties as members of a larger clinical provider group, which in turn has a contractual relationship with a specific hospital/healthcare entity to provide clinical care. In a university-based model, the relationship between individual clinicians, the academic group and the hospital is well-defined. However, the traditional university-based model is not the only employment model. In some community training settings, the relationship between individual physicians, the contract holding group and the hospital is less secure and subject to change on short notice. A sentinel case created enormous upheaval for faculty, residents and medical students and demonstrated the problems that can occur for lack of due process and a standardized approach to transitions for emergency medicine training programs.

## METHODS

The Council of Residency Directors in EM (CORD) Board of Directors formed the Faculty Due Process Task Force in 2017. The group was made up of 17 representatives from emergency medicine training programs across the country. The members were tasked to determine the key elements of due process for academic faculty and develop a position statement on due process to ensure the maintenance of high standards of excellence within training programs that undergo transitions.

Three subgroups were identified to address the ways due process affects the major stakeholders: individual faculty, residency programs, and EM trainees. Each subgroup reviewed the relevant literature and identified best practice recommendations.

### Background

Major program disruption may include administrative, financial or operational changes, or natural disasters. In 2017, a sentinel case in Ohio demonstrated that emergency medicine training programs are at risk. An academic group that administered an EM residency program since its inception lost its contract at the residency’s primary clinical site and was abruptly replaced.^1^ In addition, at the time of preparation of this manuscript, the closing of a Philadelphia hospital is currently underway, which will affect an entire EM residency program as well more than 500 other trainees.^2^ Previously, the largest hospital closure impacted approximately 350 trainees in New York City in 2010.^3^ Multiple stakeholders are affected when a major disruption occurs: the program itself, the institution’s graduate medical education (GME) enterprise (GME Committee and Sponsoring Institution), the EM trainees, as well as the patients in the community. Disruptions due to hospital finances, contract change and turnover in the faculty typically allows for some period of preparation. Due process impacts each of the involved parties, and therefore must be considered.

Major transitions as the result of natural disasters differ a bit, as they may occur without significant time for advanced planning. Hospitals, like all large institutions, are expected to have a disaster and business recovery/continuity plan. Based on our review, it is rare for these documents to address recovery/continuity of their educational mission.

## DUE PROCESS FOR INDIVIDUAL FACULTY

Individual Emergency Physicians (EPs) derive their due process rights from various sources, including the U.S. Constitution and position statements from national specialty organizations.^4^–^6^ The Fourteenth Amendment and subsequent Supreme Court rulings defined due process protections as the procedures in place when the government attempts to deprive individuals of their rights. Darlak versus Bobear (1987) was the first case to apply this concept to the medical setting. The U.S. Court of Appeals affirmed that Dr. Darlak’s medical staff privileges constituted a property interest protected by the due process clause of the Fourteenth Amendment and ruled that the hospital satisfied this obligation with hearings before the credentials committee.^7^

Physicians working outside of government institutions have other sources of due process rights. The Healthcare Quality Improvement Act of 1986 (HCQIA), which applies to all hospitals receiving federal funds, outlines fair hearing procedures for physicians and establishes immunity for members of peer review committees. The hearing requirements include: at least 30 days’ notice, a right to representation, the right to call and examine witnesses and to present evidence, the right to submit a written statement, the right to receive a written communication of the decision, and the right of appeal.^8^ Due process is also required by the Joint Commission standards.^9^ The standards include delineation of medical staff privileges and development of medical staff bylaws, along with procedures for physicians prior to having their medical staff privileges revoked. Physicians must have access to a fair hearing and appellate review.^10^

Several national physician organizations have documents that address the importance of due process protections for individual physicians. These include the Code of Medical Ethics of the American Medical Association (2007),^4^ position statements on due process from the American Academy of Emergency Medicine, (1995, 2005),^5^ and the American College of Emergency Physicians’ Emergency Physician Rights and Responsibilities (2001).^6^ Per the ACEP statement: “Emergency physicians should be accorded due process before any adverse final action with respect to employment or contract status, the effect of which would be the loss or limitation of medical staff privileges. Emergency physicians’ medical and/or clinical staff privileges should not be reduced, terminated, or otherwise restricted except for grounds related to their competency, health status, limits placed by professional practice boards or state law.”^6^

EPs have a fundamental role in patient safety. Emergency Medical Treatment & Labor Act (EMTALA) obligations ensure public access to emergency care regardless of insurance status or ability to pay. EPs are part of the safety net of emergency care and have a duty to advocate for the patient’s best interest. Physician autonomy is an essential component that enables an EP to provide safe care. EPs may face pressures regarding financial matters including admission, discharge or transfer of patients. In 2012, CBS’s *60 Minutes* special, “The Cost of Admission” details EPs pressured to perform unnecessary tests and admit a minimum number of patients.^11^ A 2016 issue of *Common Sense* details the story of a Florida emergency physician who was terminated without recourse after reporting a patient safety problem to hospital leadership.^12^ A lack of due process limits a physician’s ability to defend their actions in such cases.

In a 2013 study published in the Journal of Emergency Medicine, 62% (197 of 317) of EP respondents reported that their employer could terminate them without complete due process and 76% (216 of 284) reported that hospital administration could order their removal from the clinical schedule. Nearly 20% self-reported a “possible or real threat to employment” if they raised quality-of-care concerns.^13^

Beyond the role of patient advocate, EP faculty members also advocate for their EM trainees to help maintain educational and professional standards within their training program. In 2011, an EP was terminated without a hearing after reporting concerns of a fellow faculty member harassing female residents. In 2016, a jury found in his favor despite claims by the hospital that their actions in firing him were for “legitimate, non-retaliatory purposes.”^14^ Providing faculty with guaranteed due process protects trainees by ensuring that EPs can advocate for EM trainees without fear of termination.

There are several essential elements to due process protection for individual EPs outlined in statements from the national physicians’ organizations above. The AMA Code of Ethics stipulates the principles of a fair and objective hearing and stipulates that specialty medical societies “provide procedural safeguards for due process.”^4^ The American Academy of Emergency Medicine has detailed further that every physician is entitled to a fair hearing for adverse decisions regarding medical staff privileges, including unilateral termination by employer or other restrictions on clinical privileges. This may include revocation of medical staff membership or manipulation of clinical schedules.^5^ Due process for individual faculty is recommended by our national organizations and provides protection for faculty to voice concerns about patient safety and academic integrity.

## IMPACT ON RESIDENCY PROGRAMS AND THE GME ENTERPRISE

### Residency Program

A residency program is an entity with its own dimensions and identity, and unplanned changes can have repercussions on the program as a whole. The Accreditation Council of Graduate Medical Education (ACGME) notes that “residency is an essential dimension of the transformation of the medical student to the independent practitioner” and states that “the essential learning activity is interaction with patients under the guidance and supervision of faculty members who give value, context and meaning to those interactions.”^15^ These statements recognize that a residency program is comprised of more than a location, group of individuals, or a name.

Evaluation of the residency program is outside the scope of this paper. Instead, we focus on the effects of *en-masse* turnover of a program’s faculty in the residency program. Any large-scale turnover of faculty is disruptive. The faculty “administer and maintain an educational environment conducive to educating EM trainees in each of the ACGME competency areas.”^16^ Furthermore, faculty must also “devote sufficient time to the educational program to fulfill their supervisory and teaching responsibilities; and to demonstrate a strong interest in the education of residents,” and “maintain an environment of inquiry and scholarship with an active research component.”^17^,^18^ Every program requires a cohesive group of faculty members fully invested in education and scholarship. A primary requirement of incoming faculty must be that they possess the requisite skill set and experience to meet these expectations in order to maintain a program’s integrity.

If turnover of a program faculty does occur, the outgoing program leadership has a professional obligation to bequeath materials and processes necessary for the continued operation of the program. It would be helpful if the process for this handoff were standardized across the medical specialties. In the absence of such standardization, the faculty are left to determine which products and processes are the assets of the program and which are the intellectual property of the individual physicians. Examples of materials which are clearly in the program domain include resident evaluations, resident scholarly activities, curriculum organization, rotation goals and objectives, and Program Evaluation Committee (PEC) and Clinical Competency Committee (CCC) meeting minutes.

The incoming program must assume the responsibility for continuation of the residency program according to the ACGME Common and EM Program Requirements with little tolerance for deviation. The incoming program faculty should start with all core requirements in place and the ability to maintain the program during their tenure as program faculty.

### The Sponsoring Institution

The sponsoring institution of any program has an ethical, legal, and financial responsibility to residents and faculty of accredited programs to help ensure the stability of resources required to meet the educational mission of the training program. The ACGME has acknowledged the potential for the changing landscape of healthcare to impact residency education and as a result convened the Sponsoring Institution 2025 (SI2025) Task Force which wrote that “three forces—democratization, commoditization, and corporatization—were seen as drivers of change that appear to be guiding the future of healthcare, and thereby shaping the conditions to which GME and Sponsoring Institutions will need to adapt.”^19^ The ACGME also recognizes the importance of the sponsoring institution as demonstrated by the inclusion of hospital administrators in regular Clinical Learning Environment Review (CLER) on-site visits. The CLER Program is designed to provide hospitals and other clinical settings affiliated with the sponsoring institution with periodic feedback addressing patient safety, quality, care transitions, supervision, well-being, and professionalism. ACGME Institutional Requirements dictate that the sponsoring institution “ensure that each of its ACGME-accredited programs is in substantial compliance with the ACGME-accredited Institutional, Common and specialty-specific Program Requirements.”^20^ While major program transitions may be unavoidable, the sponsoring institution must ensure compliance with ACGME requirements and policies. During periods of transition, the highest priority is to ensure that qualified educators are in place to maintain medical education with proper supervision and minimal disruption.

The sponsoring institution is ultimately responsible for safeguarding the educational environment of a residency program despite the many contractual paradigms by which EDs are staffed. Faculty must meet educational requirements such as scholarly activity and appropriate clinical oversight even during times of transition with close monitoring by the sponsoring institution. The task force recommends the development of clear and appropriate standards; expectations and guidelines in advance of transitions will provide hospital administrators, medical administrators, program directors, staff and EM trainees with transparency during transitions. Clear educational expectations should be delineated in contract language as well as in request for proposals (RFPs); see examples in Appendices A and B.

### Graduate Medical Education Enterprise

Events that threaten the stability of a program’s faculty, leadership structure, clinical training environment, or administrative resources may also impact GME accreditation. In order to maintain the integrity of its academic mission, it is critical that each institution’s GME committee (GMEC) maintain oversight and sole governance of its training programs, similar to the self-governance of Medical Staff.

Therefore, the task force recommends that the GMEC should ideally be notified of any potential threats to the stability of a program in order to anticipate intervention and provide guidance early. GMEC involvement may prevent transition and/or help mitigate potential negative impact that may ensue. The GMEC should be consulted with appropriate notice prior to any transition to ensure that all educational needs are addressed and should be notified when a current contract is at risk of being terminated. Core faculty should never be dismissed without due process, and the GMEC should be closely involved to ensure this essential protection is not threatened. Similarly, efforts on recruitment and installation of new program oversight must involve the GMEC. The ACGME has demonstrated its willingness to suspend both Program and Institutional Accreditation if these expectations are not met at all times.

## IMPACT ON EM TRAINEES

EM residents are subject to the oversight of both the ACGME and their individual employer, which complicates their potential due process rights. From an ACGME and Residency Review Committee (RRC) perspective, EM trainees are learners. Legally, the majority are considered employees of their sponsoring hospital as well. GME funding contributes to the complexity of due process for EM residents. Federal GME funds are appropriated to hospitals, not medical schools. However, many training programs have expanded the number of residents they sponsor beyond the Centers for Medicare and Medicaid Services (CMS) cap imposed in 1997, using alternative funding including hospitals and other arrangements.^21^ Additionally, a small number of GME positions are unionized.^22^ Thus, at the individual resident trainee level, due process is dependent upon each employment scenario. In situations where residents are considered an “individual employee,” due process rights are limited. Unfortunately, most residents have little knowledge about their funding stream or their due process rights.

During major program disruption, residents are at risk due to preexisting commitments. Many have purchased homes or signed leases, have families and/or an employed spouse, children attending school, and limited financial resources, to name a few of their immediate challenges. Faculty who have been their support through EM training may now face personal employment concerns. To the trainees, communication about a transition or closure may be limited at a time when they desire transparency. These circumstances may leave the resident without clear knowledge of what to do or where to go for guidance.

This confusion may be compounded because many residents are unaware of the source of their training funds. They are also contractually bound to the residency program where they have matched, and in the event of program or hospital closure their transition to a new program is contingent upon their federal funding being released by their sponsoring institution. Funding is even more complicated for 4-year training programs, individuals with prior training or when funding comes directly from the hospital, as is the case with institutions over their CMS cap. Given the myriad of potential sources of funding for faculty positions, it is not surprising that many trainees do not understand how their EDs are staffed and under which circumstances staffing might change.

Departmental, hospital, program and GME administrators have an ethical obligation to keep residents informed of the details of an expected or ongoing major transition of staff. In the case of a potential contract changeover, trainees should be made aware of general timelines for business decisions and opportunities to initiate contingency plans. The RRC-EM should be informed in advance of the potential for program disruption to allow for an independent body to provide support and ensure clear communication to affected residents. Historically, the RRC appears to have been hesitant to get involved until change has occurred. This task force recommends a more proactive stance to better support the affected residents.

Strong, clear, and proactive hospital, departmental, and program leadership is critical. Accurate and timely information helps alleviate uncertainty. The GMEC and program leadership should work together to update residents and detail available options. While faculty will have varied availability or capability to provide advice, CORD may provide a cadre of experienced program directors to guide residents through their available options in a “just in time” fashion. A clearly identifiable point of contact to address EM trainees’ concerns is essential.

## IMPACT ON PATIENT SAFETY

Patient safety during times of transition or disaster is a primary concern. During a transition or disaster, ACGME-mandated levels of clinical supervision may be compromised to meet increased demand for emergent care of patients in need. Every effort must be made to quickly return to the accepted standard of practice, including appropriate clinical supervision. Similarly, abrupt change in faculty composition may also compromise patient care and safety. Clinical workflow processes are essential in EM and new staff may be unfamiliar with these. As EPs who are invested in residency training, faculty in emergency medicine training programs should be on the forefront of protecting both our residents and our patients. Patient and trainee safety in the clinical environment must be paramount during times of transition.

## CONCLUSION

An emergency medicine training program is a complex enterprise with multiple stakeholders. Disruptions to the educational mission include natural disasters that impact the physical training environment and wholesale faculty turnover, both of which have the potential to affect patient care and resident education. Due process protections are particularly important for individual faculty to ensure the ability to advocate for both patients and trainees. Better processes and procedures are needed to ensure the best interests of the many involved parties - the faculty, sponsoring institution, GME enterprise, trainees and patients. Clear guidelines around transitions are needed to protect the educational integrity of a training program and meet the requirements outlined by the ACGME. Improved education for residents regarding due process and GME funding issues are also essential, as we face the increasingly complex employment models that are commonplace in our specialty.

## Supplementary Information






