# Supplementary material for: The Impact of Due Process and Disruptions on Emergency Medicine Education in the United States
Source: West J Emerg Med. 2020 Jan 27;21(2):423–8. doi: 10.5811/westjem.2019.10.42800 (PMC7081866; doi:10.5811/westjem.2019.10.42800)
Supplement: Supplementary file 1 [file wjem-21-423-s001.docx]

**Appendix A**: Sample RFP with examples of clear language in support of the academic mission expected for every EM group bidding for the contract.

a. Physicians from the selected firm shall also provide clinical and didactic training to residents, house staff and medical students enrolled in educational programs conducted by ________ Medical Center.

b. Describe your firm’s experience in providing clinical and didactic educational programs for Primary Care and Specialty Care, rotating house staff, interns and medical students.

c. The program also provides an extensive program of medical education and professional training. It has several residency programs and an affiliation with the medical school and other allied health professional training centers.

d. Describe offeror’s experience in providing clinical and didactic educational programs for EM Residency Program, rotating house staff, interns and medical students.

e. Provide clinical and didactic teaching programs that meet all requirements of the Accreditation Council on Graduate Medical Education (ACGME) for interns, medical students, rotating House Staff and residents in the EM Residency Program. Designate one physician to be the E.D. Educational Coordinator, the point person for all issues regarding the teaching programs. This person may be the EM Program Director but could also be another individual such as a Vice-Chair for Education.

f. Support a setting where quality medical education and professional training are conducted for the welfare and benefit of patients and the community.

*Excerpted from the County of Santa Clara RFP ^23^*

**APPENDIX C.** Proposed Requirements for Programs Experiencing Transitions for *Incoming Program*.

II.A.1. There must be a single program director with authority and accountability for the operation of the program. The sponsoring institution’s GMEC must approve a change in program director. (Core)

II.A.2. The program director should continue in his or her position for a length of time adequate to maintain continuity of leadership and program stability.

II.A.3. Qualifications of the program director must include:

II.A.3.a) requisite specialty expertise

II.A.3.b) current certification in the specialty by the ABEM, or specialty qualifications that are acceptable to the Review Committee;

II.A.3.c) current medical licensure and appropriate medical staff appointment;

II.A.3.d) at least three years’ experience as a core faculty member in an ACGME-accredited EM program and demonstrated experience in a leadership role; and,

II.B.1. At each participating site, there must be a sufficient number of faculty with documented qualifications to instruct and supervise all residents at that location.

II.B.1.a) The faculty must devote sufficient time to the educational program to fulfill their supervisory and teaching responsibilities; and to demonstrate a strong interest in the education of residents,

II.B.1.b) administer and maintain an educational environment conducive to educating residents in each of the ACGME competency areas.

II.B.2. The physician faculty must have current certification in the specialty by the ABEM, or possess qualifications judged acceptable to the Review Committee. There must be a minimum of one core physician faculty member for every three residents in the program.

II.B.6. There must be a minimum of one core physician faculty member for every three residents in the program. (Core)

II.B.6.a Core physician faculty members must be members of the program faculty, must be clinically active and teach, and devote the majority of their professional efforts to the program.

II.B.6.b Core physician faculty members must not work clinically more than 28 hours per week on average, or 1344 hours per year, whichever is less.

II.B.6.c Core physician faculty members must include the program director and the chair/chief of EM.

II.B.8. A faculty staffing ratio of 4.0 patients per faculty hour or less must be maintained in order to ensure adequate clinical instruction and supervision, as well as efficient, high quality clinical operations.

II.C. Other Program Personnel The institution and the program must jointly ensure the availability of all necessary professional, technical, and clerical personnel for the effective administration of the program.

*Adapted from the ACGME* ^24^

**APPENDIX D.** Proposed Requirements for Programs Experiencing Transitions for *Outgoing Program*.

V.A.2.b) The program must:

(1) provide objective assessments of competence in patient care and procedural skills, medical knowledge, practice-based learning and improvement, interpersonal and communication skills, professionalism, and systems-based practice based on the specialty-specific Milestones;

(2) use multiple evaluators (e.g., faculty, peers, patients, self, and other professional staff)

(3) document progressive resident performance improvement appropriate to educational level;

*Adapted from the ACGME* ^24^
