# Supplementary material for: The Impact of Due Process and Disruptions on Emergency Medicine Education in the United States
Source: West J Emerg Med. 2020 Jan 27;21(2):423–8. doi: 10.5811/westjem.2019.10.42800 (PMC7081866; doi:10.5811/westjem.2019.10.42800)
Supplement: Supplementary file 2 [file wjem-21-423-s002.docx]

**Appendix B:** Proposed Requirements for Programs Experiencing Transitions for *Sponsoring Institution.*

One sponsoring institution must assume ultimate responsibility for the program, as described in the Institutional Requirements, and this responsibility extends to resident assignments at all participating sites. (Core)

The sponsoring institution and the program must ensure that the program director has sufficient protected time and financial support for his or her educational and administrative responsibilities to the program. (Core)

I.A.1. The sponsoring institution and participating sites must:

I.A.1.a) provide salary support or protected time for the program director;

I.A.1.b) provide salary support or protected time for each associate program director; and,

I.A.1.c) provide salary support or protected time for all core physician faculty members. Core physician faculty members must not be required to generate clinical or other income to support reduced clinical hours.

*Adapted from the ACGME* ^24^
