# Supplementary material for: The Impact of Due Process and Disruptions on Emergency Medicine Education in the United States
Source: West J Emerg Med. 2020 Jan 27;21(2):423–8. doi: 10.5811/westjem.2019.10.42800 (PMC7081866; doi:10.5811/westjem.2019.10.42800)
Supplement: Supplementary file 3 [file wjem-21-423-s003.docx]

**Table 1.** CORD Due Process Task Force recommendations for individual faculty.

1.   Individual physician groups, hospital and university employers, and other contract holders should not include due process waivers in the contracts of EPs. No provision in an EM contract shall preempt medical staff bylaws.

2.   CORD should petition the RRC to mandate due process protections for individual faculty as a requirement for accreditation of a residency program

**Table 2.** CORD Due Process Task Force recommendations for residency programs and GME enterprise.

1. Request for proposals (RFP) should include a clear language outlining the ACGME requirements necessary to successfully support the educational mission of residency training sites. This allows for protection of the residency training before a new group is even selected by ensuring, at minimum, that the new group is prepared to provide requisite resources. Sample language is noted in Appendix A.

2. The ACGME should create common program requirements for programs undergoing transition. It is imperative that the residency program be recognized as an entity with a clear stake in any hospital or faculty transition. An example of program requirements that should be adhered to during the transition is outlined in Appendices B-D.

3. The ACGME should impose a specific “transition period” for programs that experience major unexpected change such as large-scale faculty turnover.

a. This task force endorses a six-month period, during which the Sponsoring Institution and GMEC would perform frequent monitoring and assessment, including solicitation of feedback from the residents and faculty, focused on compliance with program requirements.

b. A written program update should be submitted to the ACGME-RRC, the GMEC and stakeholder groups including faculty and trainees

4. Residents should be educated about the importance of due process and its impact on their education and career. Due process should be included in both the ABEM Model of Clinical Practice and required educational curriculum within residency programs.

**Table 3.** CORD Due Process Task Force recommendations for EM trainees.

1. CORD should develop a resource than can be called upon to provide unbiased guidance/ counseling to residents and medical students during times of disaster or program transition

2. Programs should openly share faculty and trainee funding source information with residency applicants/residents. Residencies should be transparent about contracts, staffing models, payments and they should be expected to be asked about this by candidates.

3. We recommend an ACGME RC-EM policy/FAQ for disasters that addresses resident safety and support; this should include policy statements about when programs have to notify the RRC of potential upheaval when uncertainty is known (e.g. contract change, sale of hospital).

4. Hospitals and training programs should revise existing disaster response and business recovery/continuity plan to clearly outline how they will manage the institution’s educational mission.

5. Programs should have a policy that designates a point of contact within the residency and GME office in the event of a disaster or program transition.
